# Bayesian Linkage Analysis of Categorical Traits for Arbitrary Pedigree Designs

**DOI:** 10.1371/journal.pone.0012307

**Published:** 2010-08-26

**Authors:** Abra Brisbin, Myrna M. Weissman, Abby J. Fyer, Steven P. Hamilton, James A. Knowles, Carlos D. Bustamante, Jason G. Mezey

**Affiliations:** 1 Department of Biological Statistics and Computational Biology, Cornell University, Ithaca, New York, United States of America; 2 Department of Psychiatry, College of Physicians and Surgeons, Columbia University and New York State Psychiatric Institute, New York, New York, United States of America; 3 Department of Psychiatry and Institute for Human Genetics, University of California San Francisco, San Francisco, California, United States of America; 4 Zilkha Neurogenetic Institute, Keck School of Medicine, University of Southern California, Los Angeles, California, United States of America; 5 Department of Genetics, Stanford University School of Medicine, Stanford, California, United States of America; 6 Department of Genetic Medicine, Weill Cornell Medical College, New York, New York, United States of America; University of Muenster, Germany

## Abstract

**Background:**

Pedigree studies of complex heritable diseases often feature nominal or ordinal phenotypic measurements and missing genetic marker or phenotype data.

**Methodology:**

We have developed a Bayesian method for Linkage analysis of Ordinal and Categorical traits (LOCate) that can analyze complex genealogical structure for family groups and incorporate missing data. LOCate uses a Gibbs sampling approach to assess linkage, incorporating a simulated tempering algorithm for fast mixing. While our treatment is Bayesian, we develop a LOD (log of odds) score estimator for assessing linkage from Gibbs sampling that is highly accurate for simulated data. LOCate is applicable to linkage analysis for ordinal or nominal traits, a versatility which we demonstrate by analyzing simulated data with a nominal trait, on which LOCate outperforms LOT, an existing method which is designed for ordinal traits. We additionally demonstrate our method's versatility by analyzing a candidate locus (D2S1788) for panic disorder in humans, in a dataset with a large amount of missing data, which LOT was unable to handle.

**Conclusion:**

LOCate's accuracy and applicability to both ordinal and nominal traits will prove useful to researchers interested in mapping loci for categorical traits.

## Introduction

Many heritable traits, from pathogen resistance in plants [1] to panic disorder in humans [2], are described using discrete categories such as color or are quantified using discrete, ordered scales such as “mildly,” “moderately,” or “severely” affected. When performing linkage analysis of categorical traits, it is well appreciated that re-coding measurements as binary can lead to decreased power [3], [4]. Recoding measurements as continuous can lead to the same problem. Use of the most widely applied software for linkage analysis such as Superlink [5], Merlin [6], Genehunter [7], and LOKI [8] that do not employ categorical trait models is therefore not the most appropriate strategy for analyzing categorical diseases.

Most previous work done on family-based mapping of categorical traits has been restricted to particular types of pedigrees; these include backcross [9]–[11] and F2 designs [10]–[13], 4-way experimental crosses [1], [14]–[16], and sets of independent nuclear families [17]–[19]. Recent methods by Zhang *et al.* [20], Dupuis *et al.* [21], and Diao and Lin [22] allow linkage analysis for ordinal traits on arbitrary pedigrees. To date, there is no Bayesian framework for both ordinal and nominal linkage analysis on pedigrees with inbreeding loops and missing data.

In this paper, we develop a Bayesian statistical framework for linkage analysis of a categorical trait with a user-specified penetrance function of arbitrary form. We implement this framework in the software LOCate (Linkage for Ordinal and Categorical traits). Our method can analyze an ordinal or nominal trait with any number of categories, can handle missing genotype and phenotype data, and can analyze pedigrees with inbreeding loops. We demonstrate the versatility of our method's user-specified penetrance function through analysis of simulated pedigrees with a nominal trait, and find that our method outperforms LOT [20], the method of Zhang *et al.*, which is designed for ordinal traits. We further demonstrate the versatility of our method by reanalyzing a study of panic disorder in humans previously analyzed as a binary trait [2], in which many individuals have missing phenotypes. After we cut some of the pedigrees for memory considerations, our method was able to analyze the data and find evidence to reject a particular trichotomous penetrance model, while LOT was unable to handle the large amount of missing data in this study.

## Methods

In our linkage analysis framework, we seek the probability of a pedigree conditional on 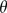, the recombination rate between a single marker locus and the unknown disease locus:
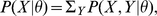
where the observed data *X* consists of individuals' phenotypes and unphased marker genotypes, and the unobserved data *Y* consists of all individuals' disease locus and phased marker genotypes, as well as any unobserved phenotypes and unphased marker genotypes. As the number of individuals in the family increases, the sum over all possible genotype assignments *Y* can grow unwieldy. Instead of considering all possible values of *Y*, Gibbs sampling is used to randomly explore the space of genotype configurations, emphasizing those configurations *Y* which have the highest values of 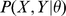, and therefore contribute the most to the summation. Below, we describe the model, demonstrate the use of simulated tempering to improve the mixing of the Gibbs sampler, and introduce a novel estimator for the likelihood of the data from Gibbs sampling.

### The model

**Figure 1:**
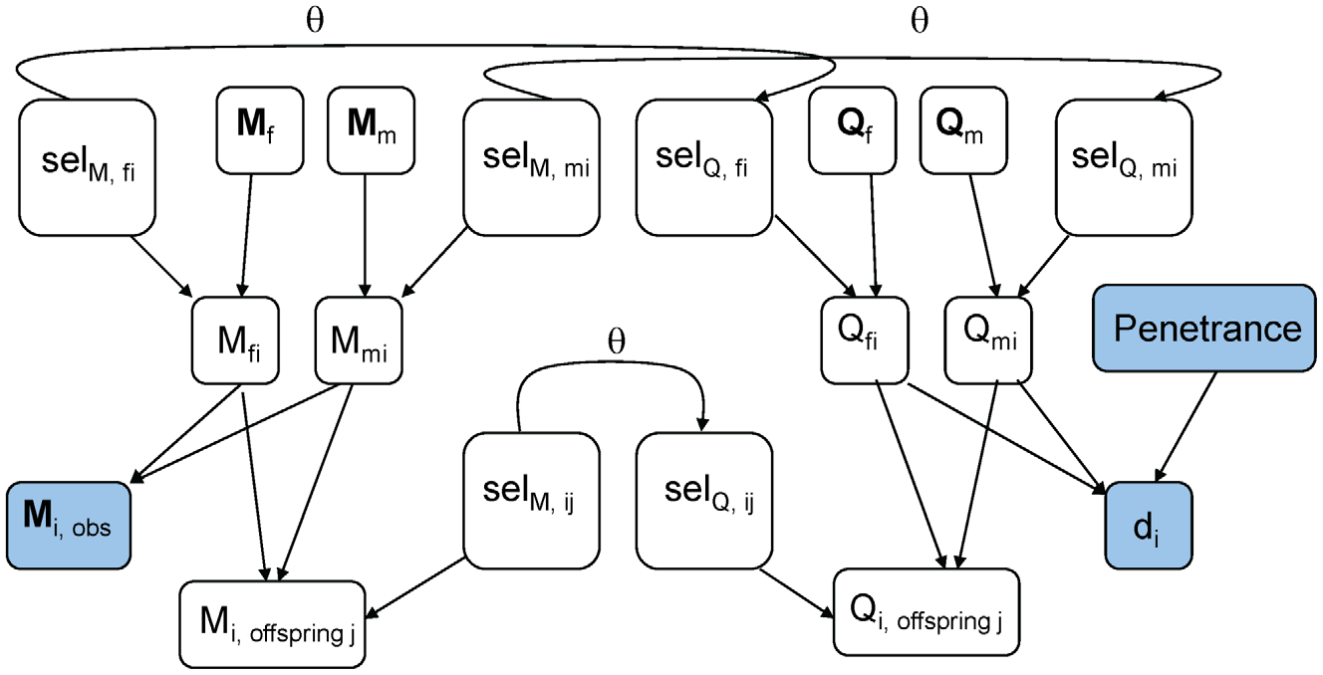
The graphical model for the Gibbs sampler. All variables shown here are involved in updating the information for individual *i*. Filled-in variables are typically observed, and held constant throughout the run of the sampler. 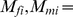
marker alleles that *i* received from its father and mother. 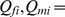
disease locus alleles that *i* received from its father and mother. 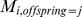, 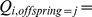
marker and disease locus alleles that individual *i* passed to its *j*th offspring. (Only one offspring is shown for illustration.) 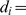
individual *i*'s phenotype. 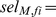
Selector variable: tells whether *i*'s paternal marker allele comes from its paternal grandfather or grandmother. 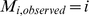
's unphased marker genotype. 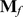, 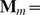
marker genotype vectors of *i*'s mother and father. If *i* is a founder, replace by a constant node describing the population allele frequencies. Penetrances = matrix of the probabilities of each phenotype, conditional on disease genotype; held constant.

Figure 1 shows the graphical model for our Gibbs sampler. Following this model, the joint probability of the observed data (*X*) and unobserved data (*Y*), conditional on the recombination rate 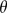, is as follows:
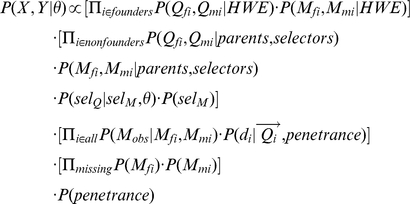
(1)where 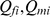 are the disease alleles individual *i* received from its father and mother; 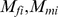 are the marker alleles *i* received from its father and mother; 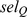 and 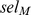 are “selector” variables that tell whether *i* received the grandpaternal or grandmaternal allele from each parent at the disease locus and the marker, respectively; 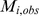 is *i*'s observed, unphased marker genotype; 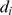 is *i*'s phenotype; and *penetrance* refers to the matrix of 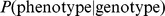 used to model the disease. *HWE* refers to the genotype frequencies assuming the founders are drawn from a population under Hardy-Weinberg Equilibrium.

We derived a Gibbs sampler to sample genotype configurations *Y* in proportion to the probability in Equation 1. In our Bayesian implementation, we used a uniform prior on the marker genotypes of individuals with missing data. We also used 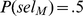, which assumes unbiased inheritance, e.g., no meiotic drive. With the availability of additional information, it would be straightforward to change these priors. The penetrance parameters, which describe the probability of each phenotype category conditional on each disease locus genotype, are assumed to have a point prior, that is, to be fixed. It would also be possible to implement a random exploration of penetrance parameters and 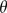 values within the Gibbs sampler; however, this would greatly increase the size of the sample space. Therefore, to maximize computational efficiency, we used a grid of values for 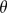 in the current implementation.

The Gibbs sampler updates each set of variables conditional on its Markov blanket [23]. The equations for the updates are given below and in Text S1. For example, individual *i*'s marker alleles and selectors 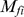, 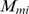, 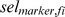, 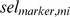 are updated by a draw from the distribution
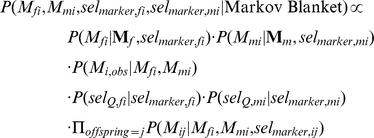
(2)where 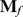 indicates the vector of marker alleles held by *i*'s father in the current iteration.

Here,
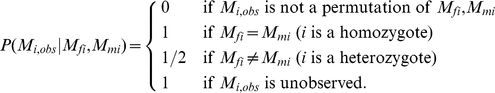
(3)In setting 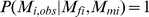 if 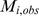 is unobserved, we assume that this individual's genotype had probability 1 of being unobserved, independent of the individual's true phased genotype. If another model for gene dropouts were available, it could be employed here.

Also,
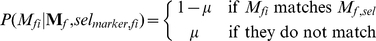
where the mutation rate 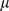 depends on the current “temperature” of simulated tempering (see below). The calculation of 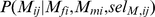 for each of *i*'s offspring is analogous to this. If individual *i*'s parents are not included in the pedigree, then *i* is a founder, and 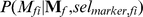 is replaced by 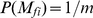, where *m* is the number of distinct marker alleles.

### Improving the Speed of the Method

Slow mixing is a chronic problem in Gibbs samplers for linkage analysis [24], [25]. This can result in inadequate exploration of the sample space and excessively long times to reach the stationary distribution. Even more of a concern is the fact that in cases with missing marker data and more than two possible marker alleles, the Markov chain may be reducible, rendering portions of the sample space inaccessible from a given starting point [25], [26].

To ameliorate this problem, we implemented simulated tempering [27], [28] in our Gibbs sampling algorithm. In simulated tempering, the Markov chain is run at several different “temperatures” 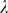, ranging from 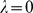, at which the chain's stationary distribution is the desired probability distribution, to 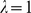, at which the chain's distribution is very “relaxed,” or smoothed, to increase the chance of the chain traversing regions of low probability density to reach different modes of the distribution. The most common way of relaxing the probability distribution is to raise the distribution to a power; however, this method is ineffective when some states to be traversed have probability zero. Geyer and Thompson [27] used an alternative approach to simulated tempering in their investigation of carrier status for cystic fibrosis in a large pedigree of Hutterites. Instead of raising the distribution to a power, they varied the disease penetrances at different values of 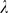. We extended their approach to a more general parameter relaxation, in which each value of 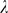 features its own penetrances, recombination rate, mutation rate, and disease-allele frequency (see Text S1). This greatly improved the mixing (Figure S5) and time to stationarity (Figure S6) of our Gibbs sampler.

### Estimating the LOD Curve

**Figure 2:**
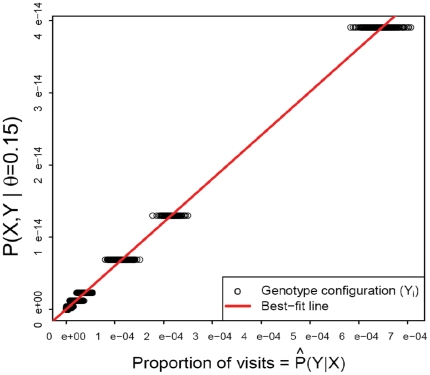
The Linear Regression estimator of 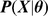. *X* = observed data, *Y* = unobserved data. 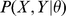 is calculated using Equation 1; 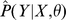 is estimated by the proportion of iterations which visit configuration *Y*, given the observed genotypes *X*. The slope of the regression line (red) is an estimate of 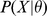.

While results of an analysis using our framework may be interpreted entirely from a Bayesian perspective by assuming a prior over the grid values of 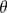, we wished to provide a log of odds (LOD) score for convenient linkage assessment. Likelihood-based parameter inference from Markov chain Monte Carlo is prone to sampling bias [26], [29]. To avoid this bias, we developed a linear regression-based estimator (LinReg) which takes advantage of the relation
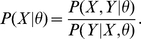
The numerator can be computed exactly (Equation 1). We estimate the denominator 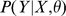 by the proportion of iterations which visit each configuration *Y*. The LinReg estimator of 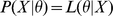 is the slope of the best-fit line (with intercept 0) through a plot of 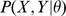 vs 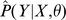, as shown in Figure 2.

### Simulations

We assessed the performance of our method using two sets of simulated data. First, we tested the accuracy of LOD score estimation for single, small simulated pedigrees. Since any errors that occur in the analysis of one pedigree will be multiplied when multiple pedigrees are aggregated in a typical linkage analysis study, it is important that our method perform accurately when only a small amount of data is available. The simulated pedigrees included from 4 to 18 individuals; some examples are shown in Figure S1. These included pedigrees with missing genotype data and with inbreeding loops. For each pedigree, we simulated either a recessive binary trait with 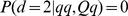 and 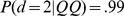, or a complete-penetrance codominant trichotomous trait (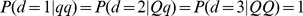). We computed the LOD scores for these pedigrees using the slightly misspecified disease penetrances in Table 1. We compared our estimated LOD scores to the theoretical LOD scores using the true penetrances, as well as to the LOD scores obtained by treating the trichotomous trait as binary (in Superlink [5]) or continuous (in Merlin [6] and SOLAR [30]). Parameter settings used for these programs are given in Text S1.

**Table 1:** Penetrance models used in our small-family simulations.

Model	Phenotype	qq	Qq	QQ
Binary	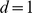	.9991	.9989	.0008
	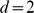	.0009	.0011	.9992
Trichotomous	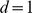	.9764	.0228	.0020
	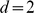	.0226	.9545	.0225
	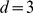	.0010	.0227	.9755

qq, Qq, and QQ represent the genotype at the disease locus.

For our second set of simulations, we assessed the ability of our method to detect linkage in cases where the pedigree(s) may be reasonably broken into a large number of small family groups or where the study includes a large number of small families. For these simulations, we considered linkage studies of 100 families, each family consisting of 2 parents and 2 offspring. We simulated a trichotomous trait with penetrances as given in Table 2 (Model A). The trait locus was either tightly linked (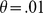) or unlinked (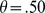) to the observed marker locus. Both the disease locus and marker locus were simulated to be diallelic, with the marker allele frequencies = .5 and the disease allele frequency = .25. We required that each simulated family be informative for linkage (at least one parent heterozygous at the marker) and exhibit at least 2 levels of the phenotype among its 4 members. We simulated 100 such studies, and examined the power vs. type I error of our method and that of LOT [20]. Because LOCate requires an estimate of the penetrances as input, we tested our method with a range of penetrances (Table 2, Models A, B, C).

**Table 2:** Penetrance models used to analyze simulated linkage studies.

Model	Phenotype	qq	Qq	QQ
A	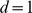	.99	0	0
	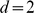	.01	.99	.01
	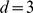	0	.01	.99
B	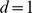	.8	.1	.1
	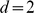	.1	.8	.1
	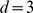	.1	.1	.8
C	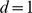	.7	.3	0
	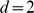	.3	.4	.3
	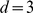	0	.3	.7

Model A was used to generate the simulations.

### Application to Data

Panic disorder is a common illness in humans, characterized by periods of intense anxiety. Because individuals exhibit varying degrees of symptoms of panic disorder, this psychiatric illness is a natural choice for analysis as an ordinal trait. We used LOCate to perform trichotomous linkage analysis on the panic disorder data set of Fyer *et al.* [2]. This dataset consists of 1591 individuals in 120 pedigrees, classified into six categories: definitely affected by panic disorder, probably affected, possibly affected, any symptoms of panic, unaffected, or unknown. The dataset has missing data among both phenotypes and microsatellite marker genotypes. Fyer *et al.* analyzed these data using ANALYZE [31] and MLINK [32], [33] using the binary penetrance model shown in Table 3, and found a two-point HLOD(.2) = 3.20 at marker D2S1788, with HLOD computed as
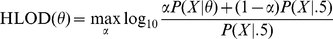
over a grid of 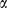 values. We reanalyzed marker D2S1788 using LOCate, under the same binary penetrance model and under four trichotomous variations of this model (Table 3 and Table S1).

**Table 3:** Penetrance models used in our analysis of Panic Disorder data.

Model	Phenotype	qq	Qq	QQ
Binary	Unaffected	.99	.5	.5
	Definite, Probable, Possible	.01	.5	.5
Trichotomous	Unaffected	.99	.5	.5
	Possible, Any symptoms	.005	.125	.125
	Definite, Probable	.005	.375	.375

In each of the variations, we used a low (.01 or .1) phenocopy rate, similar to the .01 rate used in Fyer *et al.* We varied 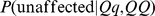 from (.5,.5) in model A, matching Fyer *et al.*, to (.05,.05) in model D, to represent a disease which is much more penetrant when individuals with “any symptoms” are included as affected.

Seven pedigrees had no observed phenotypes, due to having been collected for a different phase of the Fyer *et al.* study. Nine additional pedigrees had some observed genotypes, but were uninformative due to lack of variation in the observed phenotypes or genotypes. These pedigrees were dropped from our analysis, leaving 1332 individuals in 104 families. Of these, 35 families, ranging in size from 4 to 10 individuals, could be analyzed in LOCate on 1.7 GB-memory instances on the Amazon cloud [34]. The remaining 69 pedigrees, ranging in size from 9 to 34 individuals, would have required more than 1.7 GB of memory. We split these pedigrees into nuclear families for analysis, discarding subpedigrees which had no variation in observed phenotype or marker alleles or fewer than 2 individuals with observed genotypes, and discarding individuals without offspring which had neither observed phenotype nor genotype. After cutting, the dataset consisted of 167 pedigrees and subpedigrees, comprising 858 unique individuals.

Using LOCate, we first analyzed a reduced set of 96 subfamilies to compare 4 trichotomous penetrance models (Table S1), and then re-analyzed the full set of pedigrees using the best-fitting penetrance model (Table 3). Using multiple penetrance models is a form of multiple testing, so we must increase the LOD score threshold used to declare significance. A Bonferroni correction gives the adjusted threshold as 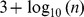, where *n* is the number of penetrance models; in this case, the threshold is 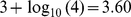. Other, less conservative approaches to correction would also be possible, such as Rom's correction [35] or determining empirical p-values by permuting phenotypes [36].

We also attempted to analyze the cut pedigrees using LOT, but found that LOT froze during this analysis. Test analyses with simulated phenotypes on the same pedigree structures revealed that this was due to the large proportion (32%) of individuals with unobserved phenotypes.

## Results

### Estimating the LOD Curve

**Figure 3:**
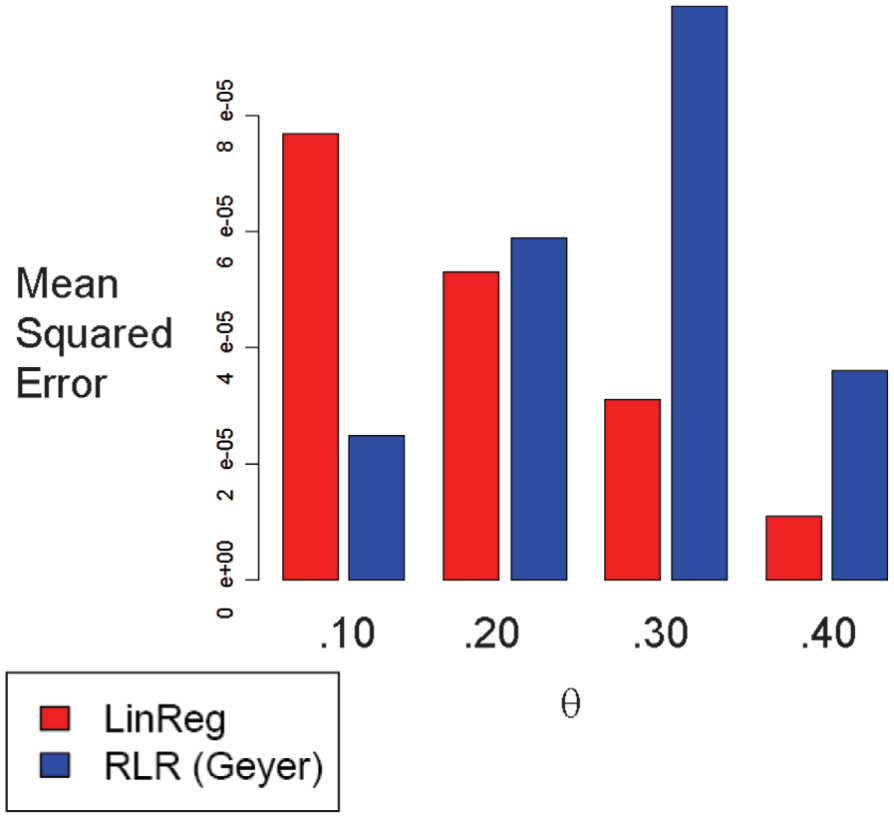
LinReg and RLR estimators of 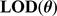. Shown are the empirical mean squared errors of the LinReg and RLR estimators of 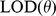 for a simulated pedigree. We used Superlink to compute the target value for each 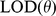.

We compared our LinReg estimator to the Reverse Logistic Regression (RLR) estimator of Geyer [37]. The LinReg estimator is faster to compute than the RLR estimator, because LinReg involves a simple linear regression, while RLR requires a complex optimization over many values of 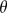. We used both estimators to estimate the LOD curve for several simulated pedigrees, for 5 different runs of our Gibbs sampler. Using Superlink to compute the exact value for each 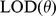, we found that the LinReg and RLR estimators have comparable empirical mean squared error (Figure 3). Given the speed and accuracy of LinReg, we used this estimator for the rest of the analyses described below.

### Simulations

**Figure 4:**
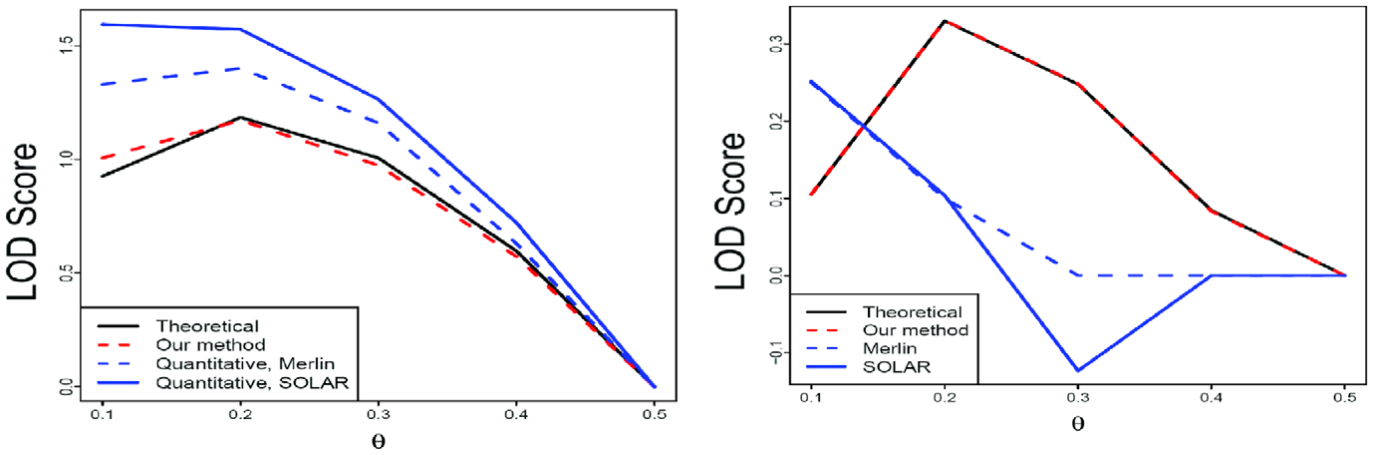
Accuracy of LOCate. Shown are the results of linkage analysis on single, simulated pedigrees with trichotomous traits. Our method (red) gives a good fit to the theoretical LOD curve (black). When the categorical trait is treated as continuous, the LOD curve estimates (from Merlin and SOLAR) are a much poorer fit (blue).

LOCate accurately estimated LOD curves for individual simulated pedigrees with binary traits (Figure S2) and trichotomous traits (Figure 4). Previous studies have shown that treating a categorical trait as binary leads to a loss of power [3], [4]. Our results concur with this (Figure S3). We also examined the effect of treating categorical traits as continuous by analyzing simulated pedigrees with Merlin [6] and SOLAR [30]. These methods' continuous-trait models were unable to estimate the LOD curves accurately, while LOCate succeeded (Figure 4). Transforming the phenotypes using Merlin's *inverseNormal* option was also not effective in improving the fit of the continuous model.

**Figure 5:**
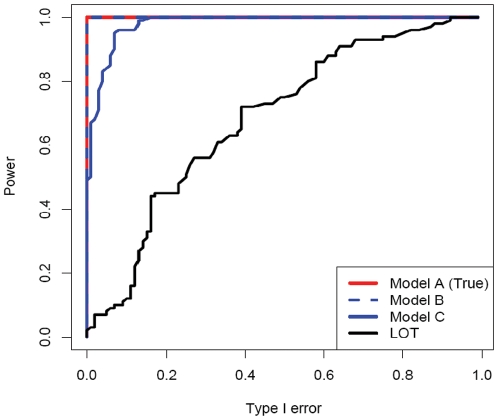
ROC plot from simulated linkage studies. Our method demonstrates better distinguishing power than LOT, even under penetrance model C, which is substantially different from model A, the values used to generate the simulations.

We present the results of our analysis of simulated 100-family linkage studies in Figure 5, which compares the receiver operator characteristic (ROC) curves for our method and for LOT. Our method has substantially higher power than LOT for the three penetrance models. Therefore, we find our method retains excellent discriminating power even when the penetrance model used is not the true model. A highly inaccurate penetrance model does reduce the magnitude of the estimated LOD scores, giving low power at a LOD threshold of 3 (Figure S4). This reinforces the value of considering alternative penetrance models in situations when LOD scores are close to zero genomewide, especially when analyzing categorical traits.

### Application to Data

The LOD scores produced by LOCate under the binary and trichotomous analyses are shown in Table 4. Although the trichotomous model we used had the highest LOD score of the four models we tested on a subset of 96 subpedigrees, when applied to all 167 pedigrees and subpedigrees this model had much lower LOD scores than the binary model. HLOD scores were also lower under the trichotomous model than the binary model (Table 4).

**Table 4:** LOD scores from our analysis of Panic Disorder data.

Model	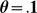	.2	.3	.4
Binary LOD	1.52	1.85	0.99	0.15
Trichotomous LOD	−10.5	−7.94	−8.08	−8.81
Binary HLOD ( 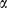 )	1.86 (0.7)	1.85 (1.0)	0.99 (1.0)	0.15 (1.0)
Trichotomous HLOD ( 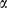 )	1.03 (0.35)	0.92 (0.5)	0.34 (0.5)	0.00 (0.0)

HLODs were computed as 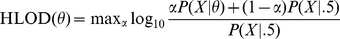. The maximizing value of 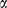 is given in parentheses.

It is clear that the necessary pedigree cutting had an effect on our results, as we found a binary HLOD(.2) = 1.85, compared to Fyer *et al.*'s binary HLOD(.2) = 3.20 on the uncut pedigrees. However, the very negative LOD scores under the trichotomous model are surprising, and give evidence that the trichotomous penetrance matrix in Table 3 is not a good model for the contribution of this locus to panic disorder. It is also possible that this locus contributes only to a broad, binary distinction between “affected/unaffected”, and that finer gradations that produce the ordinal nature of panic disorder are determined by other loci.

## Discussion

Bayesian methods for linkage analysis are useful because they allow for incorporation of prior information about allele frequencies, meiotic drive, and other factors important to linkage calculations. This, along with LOCate's versatility for ordinal and nominal traits, makes our method a valuable complementary tool to existing frequentist methods.

Even in a Bayesian framework, it is desirable to have a means of computing LOD scores, as they are commonly used to assess linkage. We developed a new, linear-regression based estimator for 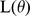, which has similar mean squared error to the RLR estimator, and is faster to compute. Our LinReg estimator will be useful for parameter inference in any situation in which MCMC is used and it is possible to calculate 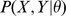, the joint probability of the observed and unobserved data, conditional on the parameter. For example, it could be used in the problem of population structure [38] to infer *K*, the number of populations represented by an observed sample of genotypes.

The choice of a penetrance model is an important question in any parametric linkage analysis, and this choice becomes even more challenging when analyzing categorical traits, as the number of possible penetrance matrices increases with the number of levels of the trait. An important distinction in the choice of penetrance matrices for categorical traits is whether the model should be ordinal or nominal. LOT estimates penetrances according to an ordinal model; this gives it an advantage for researchers who are confident their trait follows an ordinal model, but who do not wish to estimate the penetrances in advance. In contrast, LOCate is flexible to both ordinal and nominal penetrance models, but requires the penetrances to be estimated in advance. As we have done in this paper, these can be estimated on the basis of previous estimates of the phenocopy rate and overall penetrance of the trait. As our simulations demonstrate, LOCate exhibits better power than LOT when used to analyze a nominal trait, even when the input penetrance matrix is only a rough estimate. This robustness mitigates the importance of exactly estimating the penetrance matrix, and makes LOCate a valuable alternative method for researchers who wish to test penetrance models that do not have the ordinal proportional-odds property.

Due to LOCate's computational intensiveness, our simulation study was limited in scope. We believe our simulations establish LOCate as a valuable complementary approach for linkage analysis of categorical traits, particularly nominal traits. We are currently developing extensions to increase the computational speed of LOCate, which will enable a more extensive range of simulations to compare LOCate's performance to LOT on a variety of ordinal and nominal traits with varying amounts of missing data and inbreeding.

We further demonstrated the versatility of our method through a trichotomous linkage analysis of a dataset of humans affected by panic disorder with a large proportion of missing data. By splitting the most memory-intensive pedigrees into nuclear families, we were able to analyze the dataset using LOCate, while LOT was unable to process the large proportion of individuals with missing phenotypes. In this particular application, it was interesting to note the very negative LOD scores produced in the trichotomous analysis, while the binary analysis on the same set of subpedigrees had positive LODs. This demonstrates that the trichotomous model in Table 3 is a poor fit to the data. The exclusion of this penetrance matrix as a model for the contribution of D2S1788 (or a locus linked to it) to panic disorder was not possible using LOT. The exclusion of this model, a categorical “translation” of the binary penetrance model used by Fyer *et al.*, demonstrates that modeling genetic contributions to categorical traits is not a simple matter of applying a few modifications to existing binary models. Further investigation of panic disorder as an ordinal trait is needed, to establish more complete bounds on the range of possible penetrance models. In addition, further methods development, such as a Bayesian treatment of the penetrance matrix, would enable us to analyze categorical traits without specifying the penetrance matrix in advance.

We have implemented our method in the software LOCate, available at https://sourceforge.net/projects/categorical. LOCate is an effective and versatile approach for single marker analysis of nominal, ordinal, and binary traits on arbitrary family-sized pedigrees, including those with inbreeding loops and missing phenotypes and/or genotypes. While our method currently has scaling limitations for larger pedigrees, we are developing extensions for LOCate that make use of variable elimination to make the method available for multimarker analysis as well as the analysis of arbitrarily sized linkage studies.

## Supporting Information

Text S1 Equations used in variable updates, details of simulated tempering, and parameters used in other software.
(0.06 MB PDF)

Figure S1 Examples of simulated pedigrees. Black = affected; white = unaffected; gray = moderately affected. Each individual's unphased marker genotype is listed below the individual. A, B, and D are examples of simulated pedigrees with binary traits; C shows a simulated pedigree with a trichotomous trait and an inbreeding loop. Question marks in B indicate missing genotype data.
(1.74 MB TIF)

Figure S2 Estimated LOD curves for simulated pedigrees with binary traits. Our method (red) and Superlink (black) give nearly identical results.
(7.32 MB TIF)

Figure S3 Treating trichotomous traits as binary. When our method is run on simulated pedigrees with a 3-level categorical trait, the LOD curve estimate (red) is a good fit to the theoretical LOD curve (black). When the categorical trait is treated as binary, the LOD curve estimates (from Superlink) are a much poorer fit (blue).
(7.70 MB TIF)

Figure S4 LOD scores from simulated linkage studies. Red bars show the frequency of LOD scores for simulations with a linked QTL; black bars show the frequency for simulations with an unlinked QTL. Both penetrance models have good distinguishing power, but the LOD scores under the inaccurate model C have a smaller range.
(0.40 MB TIF)

Figure S5 Lag-k autocorrelation with and without simulated tempering. We show the correlation between *P(X,Y_i_)* (the joint probability of the observed and unobserved data at iteration *i*) and *P(X,Y_i+k_)* (the probability *k* iterations later). Without simulated tempering (black line), distantly separated iterations of the Gibbs sampler remain highly correlated. With simulated tempering, the autocorrelation reaches near-independence (<.05, below blue line) for *k*>15, demonstrating improved mixing of the Gibbs sampler.
(0.21 MB TIF)

Figure S6 Gelman-Rubin statistics for the likelihood of a simulated pedigree. Without simulated tempering (blue bars), the Gelman-Rubin statistics are significantly greater than 1, indicating that the chains have not reached stationarity, at a burn-in of 64,000 iterations. With simulated tempering (red bars), a burn-in of 1,000 iterations is sufficient to achieve Gelman-Rubin statistics very close to 1.
(3.49 MB TIF)

Table S1 Additional trichotomous penetrance models used to analyze Panic Disorder data. We tested each of these models on 96 subfamilies, as discussed in the Methods: Application to Data section, in addition to the selected model (model A) in Table 3.
(0.01 MB PDF)

## References

[1] Xu C, Zhang Y, Xu S (2005). An EM algorithm for mapping quantitative resistance loci.. Heredity.

[2] Fyer A, Hamilton S, Durner M, Haghighi F, Heiman G (2006). A third-pass genome scan in panic disorder: Evidence for multiple susceptibility loci.. Biol Psychiatry.

[3] Corbett J, Gu C, Rice J, Reich T, Province M (2004). Power loss for linkage analysis due to the dichotomization of trichotomous phenotypes.. Hum Hered.

[4] Feng R, Leckman J, Zhang H (2004). Linkage analysis of ordinal traits for pedigree data.. Proc Natl Acad Sci USA.

[5] Fishelson M, Geiger D (2002). Exact genetic linkage computations for general pedigrees.. Bioinformatics.

[6] Abecasis G, Cherny S, Cookson W, Cardon L (2002). Merlin - rapid analysis of dense genetic maps using sparse gene flow trees.. Nat Genet.

[7] Kruglyak L, Daly M, Reeve-Daly M, Lander E (1996). Parametric and nonparametric linkage analysis: a unified multipoint approach.. Am J Hum Genet.

[8] Heath S (1997). Markov chain Monte Carlo segregation and linkage analysis for oligogenic models.. Am J Hum Genet.

[9] Hackett C, Weller J (1995). Genetic mapping of quantitative trait loci for traits with ordinal distributions.. Biometrics.

[10] Li J, Wang S, Zeng Z (2006). Multiple-interval mapping for ordinal traits.. Genetics.

[11] Xu S, Xu C (2006). A multivariate model for ordinal trait analysis.. Heredity.

[12] Yi N, Xu S, George V, Allison D (2004). Mapping multiple quantitative trait loci for ordinal traits.. Behav Genet.

[13] Hayashi T, Awata T (2006). Interval mapping for loci affecting unordered categorical traits.. Heredity.

[14] Rao S, Xu S (1998). Mapping quantitative trait loci for ordered categorical traits in four-way crosses.. Heredity.

[15] Yi N, Banerjee S, Pomp D, Yandell B (2007). Bayesian mapping of genomewide interacting quantitative trait loci for ordinal traits.. Genetics.

[16] Yandell B, Mehta T, Banerjee S, Shriner D, Venkataraman R (2007). R/qtlbim: QTL with Bayesian interval mapping in experimental crosses.. Bioinformatics.

[17] Rao S, Li X (2000). Strategies for genetic mapping of categorical traits.. Genetica.

[18] Zhang H, Wang X, Ye Y (2006). Detection of genes for ordinal traits in nuclear families and a unified approach for association studies.. Genetics.

[19] Wang X, Ye Y, Zhang H (2006). Family-based association tests for ordinal traits adjusting for covariates.. Genet Epidemiol.

[20] Zhang M, Feng R, Chen X, Hu B, Zhang H (2008). LOT: a tool for linkage analysis of ordinal traits for pedigree data.. Bioinformatics.

[21] Dupuis J, Shi J, Manning A, Benjamin E, Meigs J (2009). Mapping quantitative traits in unselected families: algorithms and examples.. Genet Epidemiol.

[22] Diao G, Lin D (2010). Variance-components methods for linkage and association analysis of ordinal traits in general pedigrees.. Genet Epidemiol.

[23] Jordan M (2004). Graphical models.. Stat Sci.

[24] Thomas A (1994). Linkage analysis on complex pedigrees by simulation.. IMA J Math Appl Med Biol.

[25] Thomas D, Gauderman W, Gilks W, Richardson S, Spiegelhalter D (1996). Gibbs sampling methods in genetics.. Markov chain Monte Carlo in practice.

[26] Thomas D, Cortessis V (1992). A Gibbs sampling approach to linkage analysis.. Hum Hered.

[27] Geyer C, Thompson E (1995). Annealing Markov chain Monte Carlo with applications to ancestral inference.. J Am Stat Assoc.

[28] Gilks W, Roberts G, Gilks W, Richardson S, Spiegelhalter D (1996). Strategies for improving MCMC.. Markov chain Monte Carlo in practice.

[29] MacKay D, Jordan M (1998). Introduction to Monte Carlo methods.. Learning in graphical models.

[30] Almasy L, Blangero J (1998). Multipoint quantitative-trait linkage analysis in general pedigrees.. Am J Hum Genet.

[31] Kuokkanen S, Sundvall M, Terwilliger J, Tienari P, Wikström J (1996). A putative vulnerability locus to multiple sclerosis maps to 5p14-p12 in a region syntenic to the murine locus Eae2.. Nat Genet.

[32] Lathrop G, Lalouel J (1984). Easy calculations of lod scores and genetic risks on small computers.. Am J Hum Genet.

[33] Lathrop G, Lalouel J, Julier C, Ott J (1984). Strategies for multilocus linkage analysis in humans.. Proc Natl Acad Sci.

[34] Amazon Elastic Compute Cloud (2010). http://aws.amazon.com/ec2/.

[35] Rom D (1990). A sequentially rejective test procedure based on a modified Bonferroni inequality.. Biometrika.

[36] Churchill G, Doerge R (1994). Empirical threshold values for quantitative trait mapping.. Genetics.

[37] Geyer C (1991). Reweighting Monte Carlo mixtures..

[38] Pritchard J, Stephens M, Donnelly P (2000). Inference of population structure using multilocus genotype data.. Genetics.

