# Supplementary material for: Bayesian Linkage Analysis of Categorical Traits for Arbitrary Pedigree Designs
Source: PLoS One. 2010 Aug 26;5(8):e12307. doi: 10.1371/journal.pone.0012307 (PMC2928726; doi:10.1371/journal.pone.0012307)
Supplement: Text S1 — Equations used in variable updates, details of simulated tempering, and parameters used in other software. (0.06 MB PDF) [file pone.0012307.s001.pdf]

Bayesian Linkage Analysis of Categorical Traits for Arbitrary Pedigree Designs

Abra Brisbin, Myrna M. Weissman, Abby J. Fyer, Steven P. Hamilton, James A. Knowles, Carlos D. Bustamante, Jason G. Mezey

Supplementary Information

Equations used in variable updates.

The update for disease locus alleles Q_{fi} and Q_{mi}, jointly with selector variables $sel_{Q,fi}$ and $sel_{Q,mi}$, is analogous to that for M_{fi} and M_{mi} (Equation 2), with the substitution of $P(d_i|Q_{fi}, Q_{mi}, penetrance)$ for $P(M_{i,obs}|M_{fi}, M_{mi})$:

$$\begin{aligned}(Q_{fi}, Q_{mi}, sel_{Q,fi}, sel_{Q,mi} \quad | \quad & \text{Markov Blanket}) \propto \\ & P(Q_{fi} \mid \mathbf{Q}_f, sel_{Q,fi}) \cdot P(Q_{mi} \mid \mathbf{Q}_m, sel_{Q,mi}) \\ \cdot \quad & P(d_i \mid Q_{fi}, Q_{mi}, penetrance) \\ \cdot \quad & P(sel_{Q,fi} \mid sel_{marker,fi}) \cdot P(sel_{Q,mi} \mid sel_{marker,mi}) \\ \cdot \quad & \Pi_{offspring=j} P(Q_{ij} \mid Q_{fi}, Q_{mi}, sel_{Q,ij}).\end{aligned}$$

Here,

$$P(sel_{Q,fi}|sel_{marker,fi}) = \begin{cases} 1-\theta & \text{for } sel_{Q,fi} = sel_{marker,fi} \\ \theta & \text{for } sel_{Q,fi} \neq sel_{marker,fi} \end{cases}$$

where θ is the probability of recombination between the marker and the disease locus; that is, individual i's disease locus and marker alleles come from different haplotypes with probability θ.

For founders, $P(Q_{fi}|\mathbf{Q}_f, sel_{Q,fi})$ is replaced by

$$P(Q_{fi}) = \begin{cases} a & \text{if } Q_{fi} = Q \\ 1-a & \text{if } Q_{fi} = q \end{cases}$$

where a is a constant describing the frequency of the disease allele in the founder population.

If the unphased marker genotype $M_{i,obs}$ is unobserved, it is updated according to the distribution $P(M_{i,obs}|M_{fi}, M_{mi})$ (Equation 3). If the phenotype d_i is unobserved, it is updated according to the distribution $P(d_i|Q_{fi}, Q_{mi}, penetrances)$, determined by the penetrance matrix.

Simulated Tempering.

In our chain, at $\lambda = 0$, the penetrances, recombination rate, mutation rate, and frequency of the disease allele are assigned their desired values (recombination rate=θ, mutation rate=0, freq(Q) as set by user, penetrances as described in the user-specified matrix). At $\lambda = 1$, all parameters are relaxed to uniform probabilities to allow faster mixing (recombination rate=.5, disease locus mutation rate=.5, marker mutation rate=$\frac{m-1}{m}$, where m is the number of possible marker alleles; freq(Q)=.5, $P(d_i = j \mid g = k) = 1/n$, where n is the number of levels of the trait). At intermediate values of λ, each parameter p_λ is a linear combination: $p_\lambda = (1-\lambda) * p_{\lambda=0} + \lambda * p_{\lambda=1}$.

At each iteration, the temperature of the chain is updated according to a Metropolis-Hastings algorithm. The first 50,000 iterations of each sampler run are used to fine-tune the rate of temperature transitions according to the Robbins-Munro method [27]. After this fine-tuning, the chain is sampled whenever $\lambda = 0$, when its stationary distribution coincides with the desired posterior distribution $P(Y \mid X, \theta)$.

To assess whether simulated tempering was effective in improving the mixing, we examined the lag-k autocorrelation of $P(X, Y \mid \theta)$ for runs of the Gibbs sampler with and without simulated tempering, starting from the same initial configuration. Whenever the tempered chain visited $\lambda = 0$, we recorded $P(X, Y)$ for both chains. Figure S5 shows the correlation between $P(X, Y_i \mid \theta = .10)$ and $P(X, Y_{i+k} \mid \theta = .10)$ for visits i and $i + k$ to $\lambda = 0$, for $1 \leq k \leq 100$. The autocorrelation with simulated tempering (with 7 temperatures) quickly drops to below .05, "near-independence" levels, while the autocorrelation for a run of the sampler without simulated tempering remains above .3 even for $k = 100$. This demonstrates that simulated tempering effectively improved the mixing of our Gibbs sampler.

We also examined the effects of simulated tempering on the burn-in time required to reach stationarity. Figure S6 shows the Gelman-Rubin statistics we obtained for $P(X, Y \mid \theta)$ for a simulated 18-person pedigree (Figure S1D). Without simulated tempering, a burn-in time of 64000 iterations was not sufficient to achieve Gelman-Rubin statistics less than 1.05 for all values of θ; in contrast, with simulated tempering, a burn-in time of 1000 iterations sufficed, implying that the Gibbs sampler had reached its stationary distribution.

Parameter values in Superlink Online, Merlin, SOLAR, and LOT.

In Superlink Online, we performed a two point analysis with the disease allele frequency set to the simulated value, .25. When treating phenotypes $d = 1$ and $d = 2$ as unaffected, we used the recessive(.99) penetrance model. When treating phenotypes $d = 2$ and $d = 3$ as affected, we used the dominant(.99,.99) model. In both Merlin and SOLAR, we used "dummy" monomorphic markers at 11.1, 25.5, 45.8, and 80.4 cM away from the simulated marker to enable calculation of LOD scores at $\theta = .1, .2, .3$, and .4 by a Haldane map. In Merlin, we used the –assoc function, treating the trichotomous phenotype as a quantitative trait, with and without the *-inverseNormal* option. In SOLAR, we used the *multipoint* function. In LOT, we analyzed each set of 100 families together, with the disease and marker allele frequencies set to the simulated values, .25 and .5. We treated $d = 3$ as the most severe phenotype and $d = 1$ as the least severe.
