# Supplementary figures and images for: Bayesian Linkage Analysis of Categorical Traits for Arbitrary Pedigree Designs
Source: PLoS One. 2010 Aug 26;5(8):e12307. doi: 10.1371/journal.pone.0012307 (PMC2928726; doi:10.1371/journal.pone.0012307)

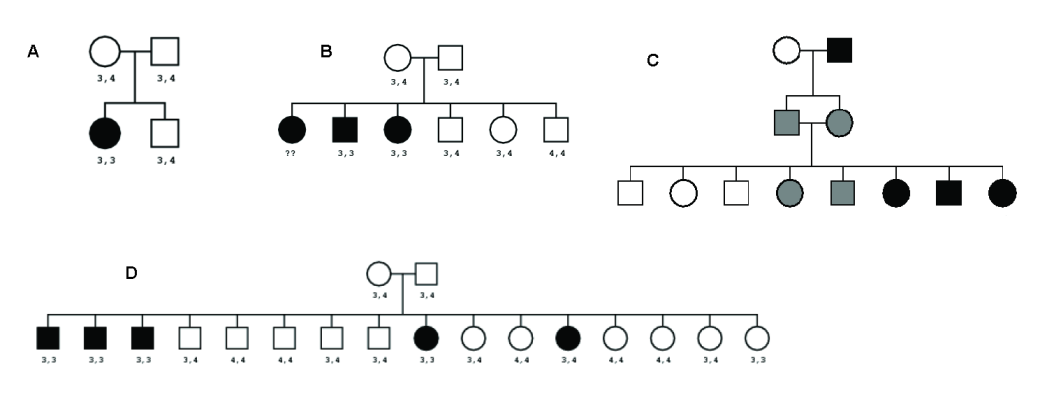

Supplement: Figure S1 — Examples of simulated pedigrees. Black = affected; white = unaffected; gray = moderately affected. Each individual's unphased marker genotype is listed below the individual. A, B, and D are examples of simulated pedigrees with binary traits; C shows a simulated pedigree with a trichotomous trait and an inbreeding loop. Question marks in B indicate missing genotype data. (1.74 MB TIF) [file pone.0012307.s002.tif]

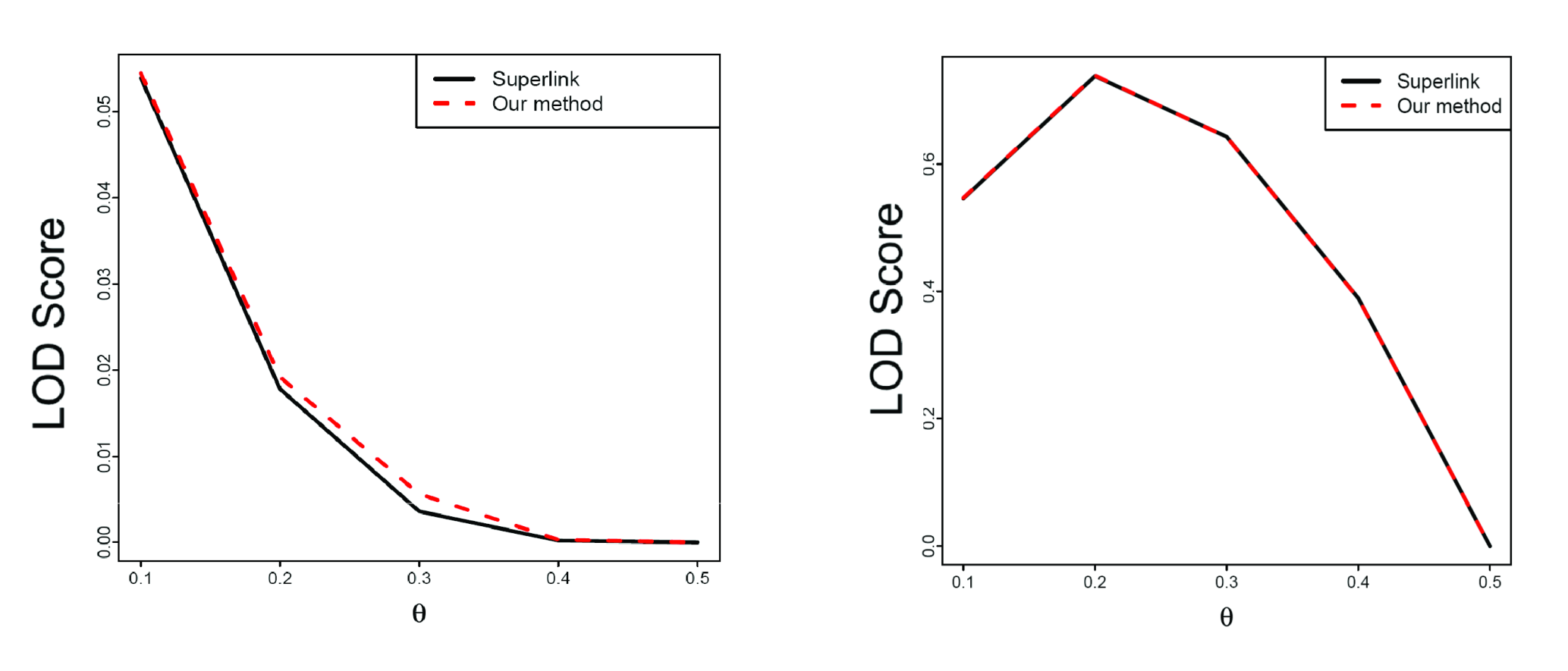

Supplement: Figure S2 — Estimated LOD curves for simulated pedigrees with binary traits. Our method (red) and Superlink (black) give nearly identical results. (7.32 MB TIF) [file pone.0012307.s003.tif]

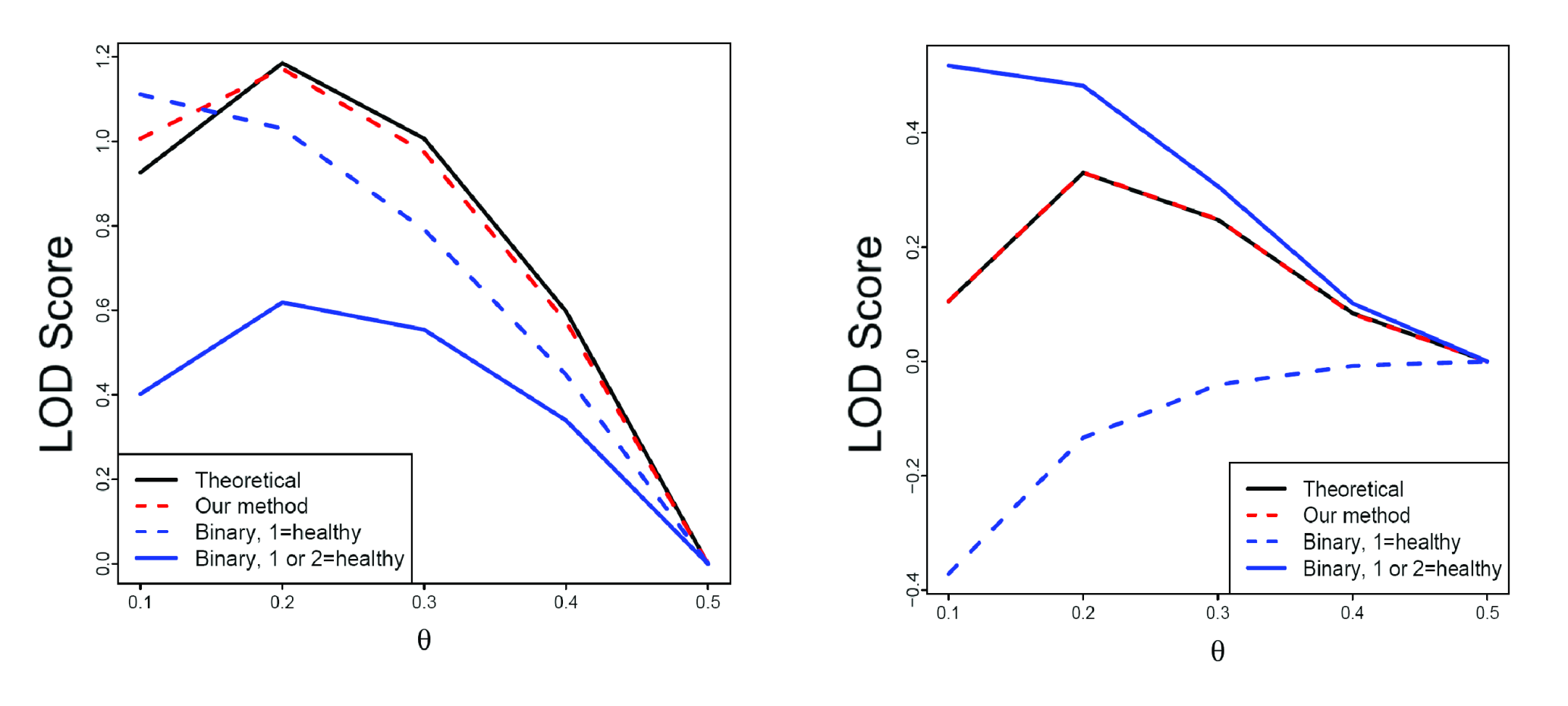

Supplement: Figure S3 — Treating trichotomous traits as binary. When our method is run on simulated pedigrees with a 3-level categorical trait, the LOD curve estimate (red) is a good fit to the theoretical LOD curve (black). When the categorical trait is treated as binary, the LOD curve estimates (from Superlink) are a much poorer fit (blue). (7.70 MB TIF) [file pone.0012307.s004.tif]

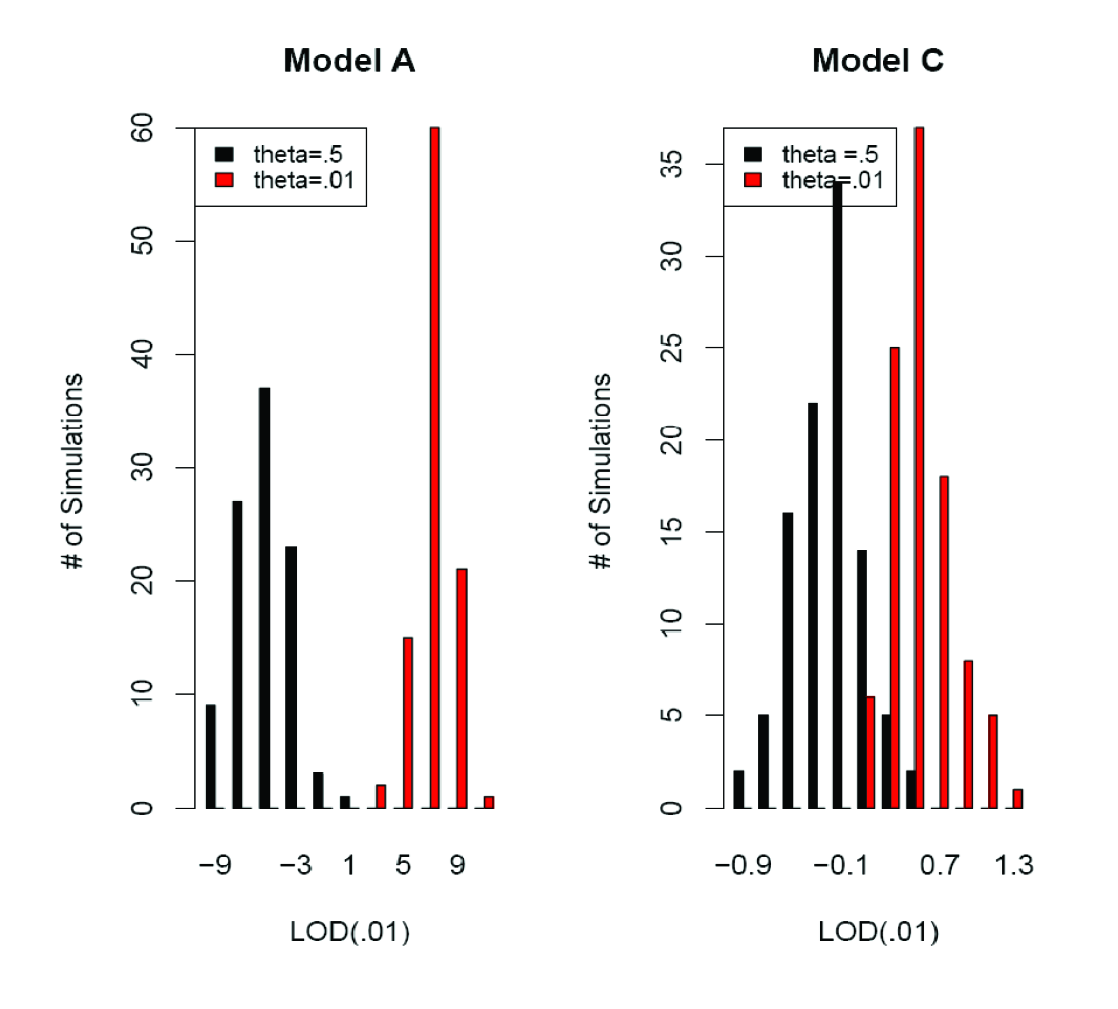

Supplement: Figure S4 — LOD scores from simulated linkage studies. Red bars show the frequency of LOD scores for simulations with a linked QTL; black bars show the frequency for simulations with an unlinked QTL. Both penetrance models have good distinguishing power, but the LOD scores under the inaccurate model C have a smaller range. (0.40 MB TIF) [file pone.0012307.s005.tif]

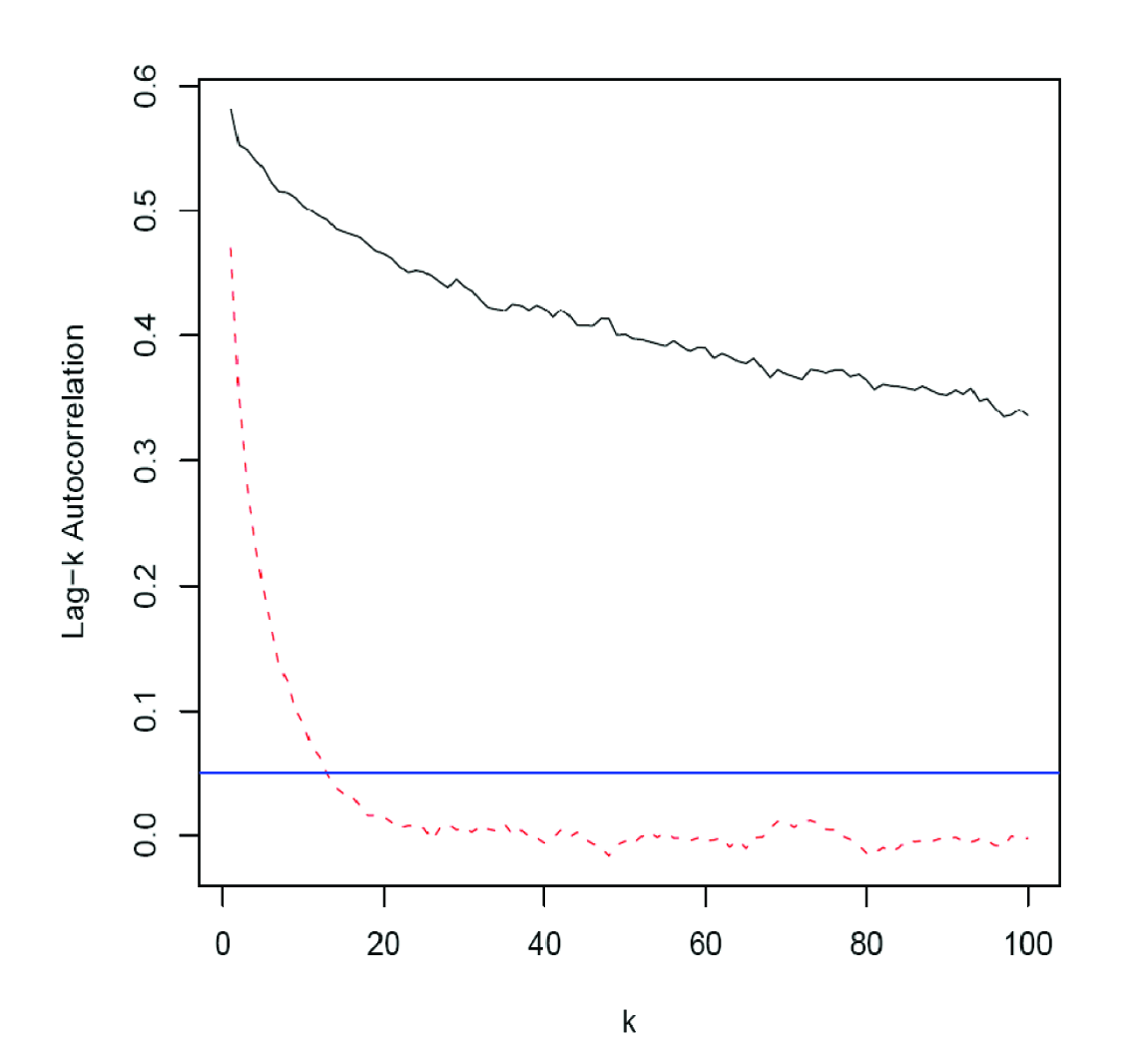

Supplement: Figure S5 — Lag-k autocorrelation with and without simulated tempering. We show the correlation between P(X,Yi) (the joint probability of the observed and unobserved data at iteration i) and P(X,Yi+k) (the probability k iterations later). Without simulated tempering (black line), distantly separated iterations of the Gibbs sampler remain highly correlated. With simulated tempering, the autocorrelation reaches near-independence (<.05, below blue line) for k>15, demonstrating improved mixing of the Gibbs sampler. (0.21 MB TIF) [file pone.0012307.s006.tif]

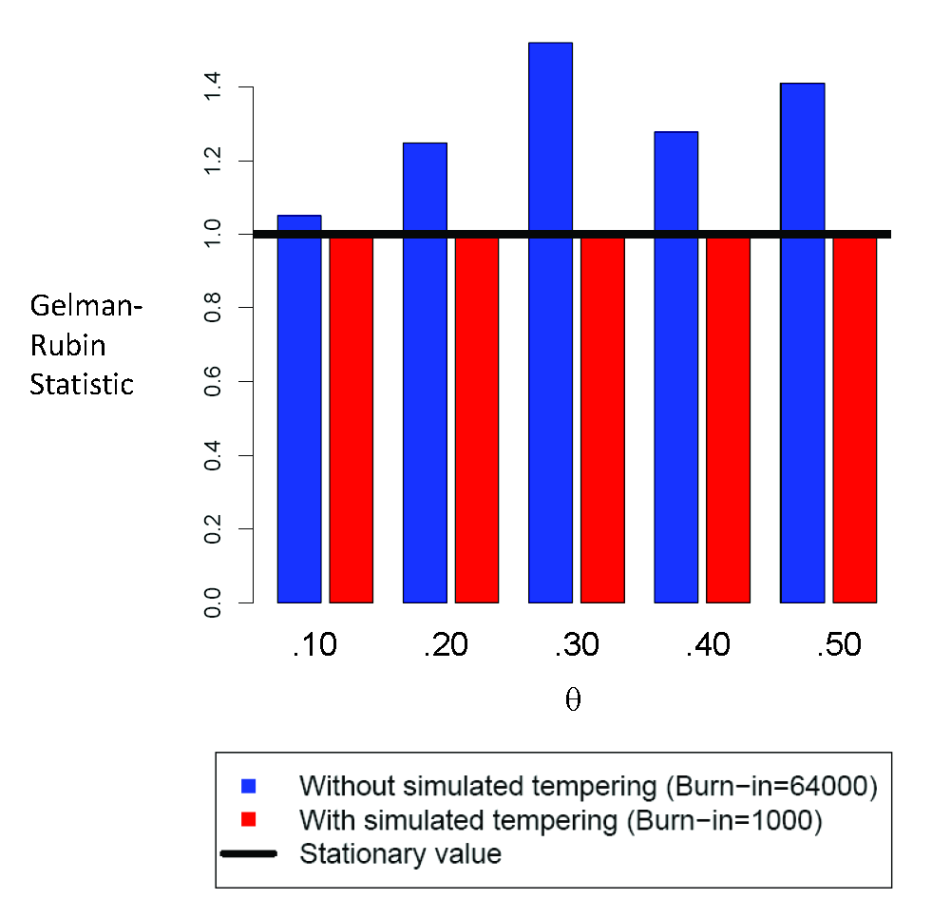

Supplement: Figure S6 — Gelman-Rubin statistics for the likelihood of a simulated pedigree. Without simulated tempering (blue bars), the Gelman-Rubin statistics are significantly greater than 1, indicating that the chains have not reached stationarity, at a burn-in of 64,000 iterations. With simulated tempering (red bars), a burn-in of 1,000 iterations is sufficient to achieve Gelman-Rubin statistics very close to 1. (3.49 MB TIF) [file pone.0012307.s007.tif]
