# Supplementary material for: Bayesian Linkage Analysis of Categorical Traits for Arbitrary Pedigree Designs
Source: PLoS One. 2010 Aug 26;5(8):e12307. doi: 10.1371/journal.pone.0012307 (PMC2928726; doi:10.1371/journal.pone.0012307)
Supplement: Table S1 — Additional trichotomous penetrance models used to analyze Panic Disorder data. We tested each of these models on 96 subfamilies, as discussed in the Methods: Application to Data section, in addition to the selected model (model A) in Table 3. (0.01 MB PDF) [file pone.0012307.s008.pdf]

Model	Phenotype	qq	Qq	QQ
B	$d = 1$	.99	.3	.2
	$d = 2$	.005	.4	.3
	$d = 3$	.005	.3	.5
C	$d = 1$	.9	.2	.05
	$d = 2$	.05	.6	.15
	$d = 3$	.05	.2	.8
D	$d = 1$	.9	.05	.05
	$d = 2$	.05	.9	.05
	$d = 3$	.05	.05	.9
